# Deep resilience: An evolutionary perspective on calcification in an age of ocean acidification

**DOI:** 10.3389/fphys.2023.1092321

**Published:** 2023-02-03

**Authors:** David A. Gold, Geerat J. Vermeij

**Affiliations:** University of California, Davis, Davis, United States

**Keywords:** calcification, evolution, fossil record, cliamte risk, holobiome

## Abstract

The success of today’s calcifying organisms in tomorrow’s oceans depends, in part, on the resilience of their skeletons to ocean acidification. To the extent this statement is true there is reason to have hope. Many marine calcifiers demonstrate resilience when exposed to environments that mimic near-term ocean acidification. The fossil record similarly suggests that resilience in skeletons has increased dramatically over geologic time. This “deep resilience” is seen in the long-term stability of skeletal chemistry, as well as a decreasing correlation between skeletal mineralogy and extinction risk over time. Such resilience over geologic timescales is often attributed to genetic canalization—the hardening of genetic pathways due to the evolution of increasingly complex regulatory systems. But paradoxically, our current knowledge on biomineralization genetics suggests an opposing trend, where genes are co-opted and shuffled at an evolutionarily rapid pace. In this paper we consider two possible mechanisms driving deep resilience in skeletons that fall outside of genetic canalization: microbial co-regulation and macroevolutionary trends in skeleton structure. The mechanisms driving deep resilience should be considered when creating risk assessments for marine organisms facing ocean acidification and provide a wealth of research avenues to explore.

## Introduction

The impact of global warming on the oceans is particularly concerning for calcifying marine species—those that build skeletons out of calcium carbonate (Orr et al., 2005). This includes a diverse swath of ocean life, including sponges, corals, molluscs, echinoderms, bryozoans, most arthropods, many red and green algae, and nanoplankton such as coccolithophores and foraminifera. These calcifying organisms are worthy of study, both for their human value—bivalves and crustaceans are critical, sustainable food sources; coral reefs are important tourist attractions—and because they represent the bulk of the fossil record (Brander et al., 2012; Narita et al., 2012; Mathis et al., 2015; Speers et al., 2016). This means any attempt to compare mass extinctions preserved in the fossil record to today’s biodiversity crisis requires an emphasis on marine calcifiers. As the oceans take up carbon dioxide from the atmosphere, the water is becoming more acidic. Basic principles of thermodynamics suggest that hydrogen ions (H^+^) present in acidified water will compete with living things for carbonate ions, making it harder for them to build calcium carbonate skeletons (Orr et al., 2005; Doney et al., 2009). When marine environments become undersaturated in calcium carbonate, acidified waters even have the potential to strip skeletons of their minerals (Pörtner, 2008). The negative effect of ocean acidification on calcifying organisms is evident in many natural and experimental case studies (Riebesell et al., 2000; Marubini et al., 2003; Michaelidis et al., 2005; Sinutok et al., 2011; Chan et al., 2012; Hughes et al., 2017). Still, it is remarkable how often living things defy the predictions of chemistry, and many calcifying organisms demonstrate little or no change in their skeletons when exposed to acidified waters (Leung et al., 2022). The skeletons of marine organisms offer a remarkable example of *deep resilience*, which we define here as the ability to maintain a consistent phenotype despite environmental change over geologic timescales. We should not forget that every living species is the product of myriad ancestors who have managed to maintain their skeletons over hundreds of millions of years, through periods where the Earth was significantly warmer than it is today, and the ocean’s chemistry was markedly less favorable to calcification (Turchyn and DePaolo, 2019; Scotese et al., 2021). In this paper, we consider experimental and historical evidence for the deep resilience of skeletons—particularly the calcium carbonate skeletons of marine invertebrates—and offer several hypotheses about the biological forces that affect this resilience. In contrast to some scientists, we doubt that the answer comes from the conservation of a genetic “toolkit”. Instead, we hypothesize that the entire organism, including its microbiome and mineralogy, must be considered to explain deep resilience. A physiological explanation is therefore required. Understanding the causes of deep resilience in skeletons will be critical to predicting the impact of climate change on marine biodiversity.

## Experimental and historical evidence for biomineral resilience to ocean acidification in marine calcifiers

### Evidence for resilience in modern settings

It is well established that marine calcifiers have unpredictable responses to acidified water. Comparative studies and metanalyses have discussed this heterogeneity in detail (Ries et al., 2009; Figuerola et al., 2021; Leung et al., 2022), so we will only touch upon it briefly. Some examples come from biogeography—natural experiments from environments where acidification is transient or constant. Upwelling, for example, is a natural process that brings CO_2_-rich deep waters up to coastal surfaces, causing rapid and extreme drops in pH (Chan et al., 2017). Yet these deep waters are also nutrient rich, and in many places, such as the California Current System, the environment is highly productive despite the increased acidity (Hofmann et al., 2014). Hydrothermal vents are another natural laboratory for low-pH environments that mimic ocean acidification. While skeletal growth and ecological diversity are diminished around vent sites (Kroeker et al., 2011; Crook et al., 2013), there are calcifiers that thrive (Uthicke et al., 2016; Connell et al., 2017). Nearly a quarter of marine species live in a habitat that is acidified or otherwise undersaturated in the magnesium-calcite needed to build skeletons (Lebrato et al., 2016). Studies of life cycle change within a single species also reveal variation in resilience. Larva, for example, generally appear far more sensitive to acidification than juvenile or adult animals (Dupont et al., 2008; Espinel-Velasco et al., 2018). Finally, there is evidence for phylogenetic differences in vulnerability to acidification, with coccolithophores, corals, sea urchins, calcifying algae, and bivalves generally being sensitive to acidification, although there are notable counterexamples in each group (Leung et al., 2022). In contrast, non-urchin echinoderms, cephalopods, bryozoans, and polychaete worms often demonstrate no change in size or calcification rate when exposed to acidified waters. Some species, notably crustaceans, even increase calcification (Ries et al., 2009; Leung et al., 2022). When considering studies that mimic near-term ocean acidification (pH ≈ 7.8 or ≈700 ppm atmospheric CO_2_), a recent metanalysis concluded that nearly 67% of species studied demonstrate no net change in growth, and ∼66% showed no net change in calcification (Leung et al., 2022). Clearly, a simple causal connection between ocean chemistry and calcification fails to predict the resilience seen in many organisms.

Many hypotheses have been generated to explain this discrepancy between prediction and observation. Behavior-based hypotheses include increases in respiration (Leung et al., 2020), and food consumption (Ramajo et al., 2016), which could counteract the energetic costs of maintaining shells in acidified waters. Evolutionary explanations include population hybridization and epigenetic changes in gene regulation. Simple natural selection could explain some aspects of resilience, as high levels of larval mortality following exposure to acidified water can shift genetic diversity in the adult population (Pespeni et al., 2013; Cornwall et al., 2020). Larval mortality is typically high and could play an important role in the rapid adaptation of organisms to ocean acidification, assuming that there is suitable genetic variation (Sunday et al., 2011; 2014; Bitter et al., 2019). All these mechanisms likely play some role in the resilience of skeletons in marine calcifiers. However, it is not clear that any of these can explain long-term patterns seen in the fossil record, which records evidence of deep resilience.

### Evidence for deep resilience in the fossil record

The concept of deep resilience is derived from deep (geologic) time, and cannot be inferred by studying living organisms alone. There have been some attempts to connect climate change and species success over (geologically) short-term timescales. For example, stony coral fossils contain isotope signatures that serve as a proxy for ocean temperatures, meaning coral distributions and climate can be correlated over hundreds of thousands of years (Edmunds et al., 2014). Our paper focuses on even longer timescales, looking at patterns over hundreds of millions of years.

There are several patterns from the fossil record that speak to the long-term resilience of skeletons. Firstly, once a group of related organisms (a clade) evolves a mineralized skeleton, it is very unlikely that the basic mineralogy changes over evolutionary time. Most skeleton-building clades trace their ancestry back to the Cambrian, ∼539–485 million years ago (Ma). In addition to many clades evolving calcium carbonate skeletons, others evolved skeletons made of calcium phosphates or silicates. For those with calcium carbonate skeletons, most clades settled on one of two polymorphs—calcite or aragonite. Both calcite and aragonite are made of the same molecule, but they are packed into minerals in different ways, and have different properties related to solubility, strength, and the degree in which magnesium can be incorporated as an alternative to calcium. Whether a group evolved a skeleton of calcite or aragonite correlates to which of the two polymorphs precipitated as the dominant mineral in the ocean at the time each skeleton originated (Porter, 2007). This suggests that the environment was an important influence in the mineralogy of the earliest skeletons. Counterintuitively, after the first skeletons evolved, their mineralogy rarely changed over deep time, even when the environment no longer favored that particular mineralogy (Murdock, 2020). The oceans have switched back and forth between calcite-dominated waters and aragonite-dominated waters several times over the last 500 million years (Sandberg, 1983), yet these switches are not reflected in the skeletons of organisms. Instead, those with calcite skeletons continue to make calcite skeletons—and aragonitic organisms make aragonite skeletons—regardless of the abundance of that polymorph in the oceans. Some interesting exceptions come from species with bimineralic skeletons, those that incorporate calcite and aragonite in their shells, but again, most of these clades evolved the ability to incorporate both polymorphs early in their evolutionary history (Murdock, 2020). The lack of change in skeletal mineralogy despite changes in ocean chemistry is one example of deep resilience.

Another example of deep resilience comes from the apparent decoupling between skeleton mineralogy and extinction risk. When considering “background” extinctions (excluding the five mass extinction events in the fossil record), there is a general, long-term decrease in extinction rates over time (Raup and Sepkoski Jr, 1982; Foote, 2000; Alroy, 2015; Kocsis et al., 2019; Kröger et al., 2019; Stockey et al., 2021). Specifically, extinction rates are elevated in the Cambrian and Ordovician (∼485–444 Ma) and drop dramatically afterwards. The cause of this pattern is unknown, although a recent paper proposes a connection to atmospheric oxygen levels (Stockey et al., 2021). Regardless of cause, this suggests that Earth’s organisms went through an early bottleneck, where those less resilient to environmental perturbations were eliminated. It is unclear whether skeletal stability was an important factor in early extinctions, though there is a known relationship between biomineralization and oxygen consumption (Towe, 1970; Stockey et al., 2021). An intriguing pattern that further supports the connection between skeletal form and extinction risk is that greater variation exists in Cambrian/Ordovician skeletons than seen afterwards. Examples of skeletons that failed to survive through the end-Ordovician include: the enigmatic “small shelly fossils” of the early Cambrian, many of which were mineralized with calcium phosphates and silica (Bengtson, 2004); Cambrian sponges with spicules featuring a mixture of silica and calcium carbonate (Botting and Butterfield, 2005; Botting et al., 2012); the hyolithohelminths and Byroniida that built phosphatic tubes (Kouchinsky et al., 2012); and phosphatic arthropods such as bradoriids, phosphatocopids, and aglaspidids (Zhang et al., 2011; Kouchinsky et al., 2012; Lerosey-Aubril et al., 2013). In his review on animal biomineralization, Murdock. (2020) demonstrates that, even in clades where mineral form has remained stable, the details of mineralogy were more disparate in the Cambrian than today. It is possible that the patterns described in this paragraph are mere correlation, and it is important to emphasize that the long term decline in extinction is not restricted to mineralized organisms (Raup and Sepkoski Jr, 1982; Foote, 2000; Alroy, 2015; Kocsis et al., 2019; Kröger et al., 2019; Stockey et al., 2021). Still, the patterns are consistent with the hypothesis that those lineages which survived the turbulent early phase of the Cambrian/Ordovician were more resilient than their extinct peers.

A more direct line of evidence for skeletal resilience comes from Eichenseer et al. (2019), who examined how calcitic and aragonitic clades responded during shifts between calcite and aragonite-dominated oceans. From the Ordovician to the Middle Jurassic (∼485–174 Ma) a positive, linear relationship exists between ocean chemistry and the abundance of marine species using the dominant calcium carbonate polymorph in their skeleton. No relationship is found from the Late Jurassic onwards. The authors attribute this decoupling of clade abundance and ocean chemistry to the diversification of calcifying nannoplankton, which helped buffer the marine carbonate system. Prior to the Jurassic, marine carbonates were primarily deposited on continental shelves, meaning the amount of carbonates sequestered in the ocean was highly sensitive to the position of the continents at any given geologic time (Ridgwell and Zeebe, 2005). But starting in the late Triassic, several groups of phytoplankton evolved biomineralization, heralding a new chapter in resilience. Calcification in plankton provides a range of benefits, including protection against grazing, photodegredation and viral attack (Monteiro et al., 2016). A radiation of mineralized coccolithophores, diatoms, and foraminifera though the Jurassic meant that more ocean carbonates were being sequestered into nanoplankton skeletons, and when such plankton died their skeletons acted as ballast, increasing carbonate deposition into the deep open ocean (Ridgwell and Zeebe, 2005; Eichenseer et al., 2019). This production of a deep sea carbonate sink correlates with greater stability of saturation state in the carbonate record, and demonstrates how the evolution of new calcifying organisms could improve biological resilience over geologic time. We suspect there are additional factors driving resilience in non-planktonic organisms, some of which we will discuss at the end of this paper.

Regardless of cause, a pattern of post-Jurassic resilience remains. No type of skeletal mineralogy has been completely lost to extinction since the Jurassic, and post-Jurassic periods of intense global warming do not correlate with elevated extinctions for calcifiers. For example, large declines in both calcium carbonate and calcifying nanoplankton during global warming events in the Jurassic/Cretaceous have previously been interpreted as evidence for a “biocalcification crisis'', but new evidence suggests this is actually a preservation bias (Slater et al., 2022), and that nanoplankton demonstrated resilience. Instead of loss, new groups developed mineralized skeletons during these warm periods. Examples include two groups of polychaete annelid worms, the tube-building serpulids and sabellids, some of which mineralize even in the highly undersaturated waters of the deep sea (Taylor and Vinn, 2006; Vinn et al., 2008; Kupriyanova et al., 2014; Kupriyanova and Ippolitov, 2015). Another historical example of intense warming is the Paleocene-Eocene Thermal Maximum, where global temperatures increased ∼5 °C over ∼170,000 years due to methane degassing (though considerable debate remains regarding the duration, rate, and intensity of ocean warming at this time) (Charles et al., 2011; Gutjahr et al., 2017). This resulted in the migration of nannoplankton and elevated extinctions of foraminifera (Alegret and Ortiz, 2006), but otherwise there are no notable marine extinctions (Keller et al., 2018). This general decrease in extinction risk over time offers a final historical line of evidence for the resiliency of marine calcifiers.

Taken together, a compelling case for deep resilience can be inferred from the fossil record. Early in evolution, there was greater variation in skeletal mineralogy than there is today. Many of these forms were lost to extinction through the Cambrian and Ordovician, and the groups that survived waxed and waned depending on whether ocean chemistry was in their favor. But over hundreds of millions of years those species that survived demonstrated less sensitivity to the environment, with evidence of a major decoupling by the mid-Jurassic. Ocean chemistry and temperature have fluctuated dramatically since then, yet only one mass extinction is recognized in the marine fossil record, which was caused by an extraterrestrial impact that is a poor analogy for modern climate change (Keller et al., 2018). These patterns in the fossil record offer striking evidence for deep resilience, but the mechanism underlying this pattern is unknown.

## Canalization of a “biomineralization toolkit” does not explain deep resilience

A decrease in phenotypic variability over geologic time is not unique to skeletons; instead it is one example of a broader trend in canalization. Canalization describes a phenomenon observed in ontogenic development (i.e. the growth of a fertilized egg into an adult) where individuals robustly produce physical traits regardless of variation in the environment or the underlying genes (Waddington, 1962). The concept of canalization has been used to explain resilience of form in the fossil record, particularly though the hypothesis that development was less canalized in the deep past than it is today (Valentine, 1995). As evolution proceeds, more and more genetic pathways are thought to be layered onto the developmental process, making it increasingly difficult to modify the original pathway without causing damaging**,** unintended side-effects. Canalization was integrated with the concept of gene regulatory networks by Davidson and Erwin as an attempt to explain the resilience of animal bodyplans since the Cambrian (Davidson and Erwin, 2006; 2009). In this retelling, the addition of network “subcircuits” over evolutionary time adds to the complexity of the system, making it harder for drastic modifications to occur. MicroRNAs and other forms of genetic regulation add additional layers of complexity, increasing the stability of developmental processes (Peterson et al., 2009). Such a scenario could potentially explain the patterns in skeletal evolution summarized in the previous section. Murdock (2020) explicitly endorses such a hypothesis, stating “[t]he first skeletal tissues in animals show greater disparity than their descendants, being subject to looser biological control prior to canalization.” In their excellent review of biomineralization, Gilbert et al. (2022) similarly conclude that the elucidation of gene regulatory networks is one of the most promising future directions on the subject. While reconstructing gene networks is an important task for understanding the biology and evolution of skeletal formation, we are skeptical that such exercises will explain the deep resilience of skeletons. This has to do with an emerging paradox between the similarities of skeletons and a lack of conserved genes.

The concept of genetic canalization is closely linked with (though not synonymous to) the search for a “biomineralization toolkit”—or a set of conserved genes that underlie biomineralization across different organisms. Presumably this toolkit would contain the core network of genes that became canalized over the course of evolution. Scientists broadly agree that mineralized skeletons evolved dozens of times, so the toolkit is not thought to be inherited from an ancestral, skeleton-bearing organism (Knoll, 2003; Murdock, 2020). Instead, it is hypothesized that an ancient mechanism to metabolize and manage mineral buildup was co-opted by many different lineages to quasi-independently generate the first skeletons. If this hypothesis is correct, we would anticipate finding similar sets of genes used during biomineralization, and that many of these genes should also be found in lineages that do not mineralize skeletons. This basic pattern has been confirmed to some degree by gene expression studies, which focus on the transcription factors that regulate biomineralization, as well as proteomic studies looking at the structural proteins that scaffold skeleton minerals (Drake et al., 2013; Mass et al., 2013; Ramos-Silva et al., 2013; Peled et al., 2020; Mummadisetti et al., 2021; Gilbert et al., 2022). Some conserved candidate genes include carbonic anhydrases, tyrosinases, SPARCs (Secreted Protein Acidic and Rich in Cysteine) as well as genes coding for von Willebrand factor, Sushi/SCR/CCP, and laminin protein domains. Yet there are several reasons to be cautious about over-interpreting this list:

Firstly, there is a bias in comparative genetics to focus on similar genes. Studies that leverage transcriptomics to compare species at the ordinal taxonomic level or higher tend to find little overall conservation; those that attempt to quantify similarity place the number of conserved transcripts at ∼15% or less (Jackson et al., 2010; Luo et al., 2015; Aguilera et al., 2017; Conci et al., 2019). Similar results have even been found at finer taxonomic levels. For example, a recent study comparing mantle tissue transcriptomes from five species of the mussel genus *Mytilus* identified 552 conserved genes out of 6,130, or ∼9% (Malachowicz and Wenne, 2019). The study of structural proteins provides similar results. The currently hypothesized “core biomineralization toolkit” for stony corals—those proteins shared across all analyzed species—consists of a mere six (Zaquin et al., 2021). Species-specific co-option of independent proteins appears to best explain the vast differences between their organic matrices (Zaquin et al., 2022). The resolution in these comparative studies is limited given the small number of species studied so far, but current data suggests that conserved genes make up a fraction of the genes involved in biomineralization.

Secondly, most of these “conserved” genes are actually members of large gene families, which demonstrate similarities in certain protein domains but are otherwise dissimilar. These genes are notable for containing repetitive, low complexity domains, which evolve rapidly, and have a proclivity to expand, contract and rearrange in the genome through domain shuffling (Ramos-Silva et al., 2013; Kocot et al., 2016; Aguilera et al., 2017). This makes it very difficult to move past general claims of gene “similarity” between organisms and to identify *homologs*—genes in different species that descend from a single, ancestral gene. Without evidence that genes from different species are homologs, there is little reason to assume their common ancestor used such genes in the same way for their own skeletons. Linguistic gymnastics conflating “similarity” and “homology” are rife in the biomineralization literature. One study comparing the closely-related freshwater mussels *Elliptio complanata* and *Villosa lienosa* found 31 out of 48 “similar” proteins in the nacre organic matrix (they did not attempt to determine homology) and claimed “A few of these proteins … appear to be analogues, if not true homologues, of proteins previously described from the pearl oyster or the edible mussel nacre matrices, thus forming a remarkable list of deeply conserved nacre proteins” (Marie et al., 2017)*.* What these studies appear to attest to is not the deep conservation of a genetic toolkit, but a remarkable ability for organisms to continually recruit and remix genes that structure their skeletons.

The mixed results from comparative genetics is exemplified by echinoderms. Perhaps the best studied gene regulatory network comes from the larval endoskeleton of the sea urchin *Strongylocentrotus purpuratus* (Rafiq et al., 2014). Scientists broadly agree that the calcitic stereom skeleton unique to echinoderms originated in a common ancestor, although the skeleton has been gained and lost in different life stages over the course of evolution (Bottjer et al., 2006). Yet comparative work looking for S. *purpuratus* genes in the skeletons of other echinoderms has yielded mixed results. Of the 38 vetted proteins identified in the skeleton of the brittle star *Ophiocoma wendtii*, only 26 demonstrated similarity to proteins found in *S. purpuratus*, and none could be identified as clear homologs (Seaver and Livingston, 2015). MSP130, one of the most abundant proteins in the *S. purpuratus* skeleton, is notably absent. In another brittle star (*Amphiura filiformis*), the authors recovered 23 of 56 *S. purpuratus* biomineralization genes, and only one of 14 genes specifically involved in the mineralized spicule matrix (Dylus et al., 2018). In the sea star *Patiria miniata*, 85 proteins were identified in the skeletal proteome; 36 had homologs in *S. purpuratus* with another 29 showing sequence similarity (Flores and Livingston, 2017). MSP130 was not identified in the *P. miniata* skeleton, neither were proteins with C-lectin domains and/or acidic repetitive regions, which are common in sea urchin and brittle star skeletal proteomes. All of these papers endorse the hypothesis that the gene regulatory network described in *S. purpuratus* has undergone extensive reorganization in these other taxa. This supports an emerging hypothesis that the “effectors” of echinoderm skeletogenesis—genes which are downstream of the core network but play a more direct role in biomineralization—exhibit rapid evolution (Shashikant et al., 2018).

A final, illustrative example of the limitations of the “biomineralization toolkit” concept comes from the recent *Natilus pompilius* genome (Zhang et al., 2021). The paper focuses on shell matrix proteins, which guide the growth of calcium carbonate in the shell. The authors identified 78 shell matrix proteins using gene expression analysis of the mantle tissue, and compared their list to previously described shell matrix proteins in a gastropod (*Lottia gigantea*) and several bivalves (*Crassostrea gigas*, *Mya truncata*, and *Pinctada fucata*). Of those 78 genes, 21 “similar” shell matrix proteins could be found in at least one of the other four mollusc species. These were similar in the sense that the proteins share conserved domains such as Sushi/SCR/CCP, laminin, chitin-binding, and carbonic anhydrase domains. Yet the most enriched shell matrix proteins in *N. pompilius* lacked any conserved domains known from other species. The authors used the program OrthoFinder (Emms and Kelly, 2015), to identify homologs among the five species. 52 of 78 shell matrix proteins were specific to *N. pompilius*. No combination of species resulted in more than six conserved homologs. Even the protein Nautilin-63, which was previously found to be a key player in the shell matrix of *Natilus macromphalus* (also known as *Allonautilus macromphalus*), is highly dissimilar to the *N. pompilius* homolog. To determine how dissimilar they are, we used MUSCLE (Edgar, 2004) to perform a quick alignment of *N. pompilius* Nautilin-63 (EVMG013998.1) and the *A. macromphalus* homologue (NCBI accession: P86702); we found that a mere 14% of amino acids were identical between the proteins of these two species. It appears that little of the molecular underpinnings of nautilus shell formation could qualify as part of an ancient, shared “toolkit.”

To conclude, we are not arguing that there is no such thing as a biomineralization toolkit, or that molecular studies of biomineralization are unimportant. Most of the evidence discussed here relates to the shell matrix proteins, which would presumably be the end-members of a biomineralization gene regulatory network. It is plausible that a conserved gene regulatory network, currently undiscovered, is involved in partitioning and recruiting these shell matrix proteins. In fact this is what proponents of the gene regulatory network model would predict (Davidson, 2010). Testing this will require detailed functional studies on individual species to resolve their gene regulatory networks. And there is plenty of room for a ‘biomineralization toolkit’ in the sense that a common set of ancestral genes were independently co-opted for building skeletons in many groups. But the patterns seen in the genetics of skeletal formation contradict the predictions of canalization. Instead of genes getting “locked” into place, biomineralization appears to be a prime example of rapid evolution, with new genes being replaced, co-opted, modified, and shuffled at such speed that identifying clear homologs is near impossible. This suggests to us that the deep resilience seen in the fossil record cannot be explained by genetic canalization, and that other mechanisms play a causal role.

### Alternative mechanisms of deep resilience

If genetics cannot explain deep resilience in calcifiers, what can? We conclude this paper with a consideration of two mechanisms that might help—macroevolutionary trends in skeleton structure and microbial interactions—and consider some future directions of study.

### Macroevolutionary trends in skeleton structure

For animals whose skeletons offer protection from the environment, it is well established that skeleton evolution has been shaped by the selective pressures of predators (Vermeij, 1987). The radiation of generalist, skeleton-breaking (durophagous) predators, beginning in the Mesozoic, led to a range of changes in the morphology and behavior of prey. This phenomenon is best-studied in the molluscs, where predatory escalation resulted in the increased thickness of shells, the elaboration of spines and whorls, and possibly a reduction in the amount of organic matter in shells over geologic time (Vermeij, 1987; Vendrasco et al., 2018). That last trend appears to have been driven by the replacement of many early molluscs, whose shells featured loosely organized bundles of calcium carbonate fibers in an organic matrix, with clades whose shells featured stronger textures such as nacre and crossed lamellar structures (Vendrasco et al., 2018; Murdock, 2020). This could offer another example of the evolutionary “bottleneck” described earlier, where the lineages that have survived to the present are those that feature more resilient skeletons. Outside of the molluscs, other possible impacts of predation on marine calcifiers includes the evolution of thickened and “irregular” skeletons in echinoids, the expansion of encrusting species with protective exoskeletons like serpulid worms and bryozoans, the radiation of coralline algae and scleractinian corals, and the independent evolution of burrowing/boring behaviors (Wilson and Palmer, 1990; Wood, 1995; Walker and Brett, 2002). Although the primary agent of selection is predator/prey escalation, these novel forms are likely to demonstrate greater resilience more generally. We hypothesize that two predatory responses in particular—drilling predation and bioerosion—have shaped changes in skeleton structure in ways that could help explain deep resilience.

Drilling is a means of predation on hard-shelled animals practiced principally by gastropods, which use acidic secretions from the hypobranchial gland to excavate a perforation in the shell wall through which the predator inserts the proboscis in order to consume the prey’s soft parts. Although drilling has a long history dating back to the first biomineralized skeletons in the Late Ediacaran (∼635–539 Ma), it became increasingly important as a cause of death and as an agent of selection following the evolution of naticid and muricid gastropods during the Mesozoic (Vermeij, 2015; Klompmaker et al., 2017). These drillers largely preyed on molluscs and barnacles, whereas cassids, which largely evolved during the Cenozoic (∼66–0 Ma), prey on echinoids (Petsios et al., 2021). Changes in skeletons brought on by this Mesozoic marine revolution could provide an additional mechanism to help explain the decoupling of animal biomineralization and environment post-Jurassic (Eichenseer et al., 2019). Prey species have evolved numerous adaptive responses to drilling, including features of shell sculpture, shape and thickness, but from the perspective of the present paper the most important are mineralogical and microstructural traits. One example is the organic sheets separating layers of mineral in the shell (Harper, 1994; Ishikawa and Kase, 2007). Many balanomorph barnacles, for example, evolved organic sheets and tubes within plates (Buckeridge et al., 2008). Studies have shown that molluscs and barnacles can modify their shell microstructure in response to drilling and other forms of predation, indicating a degree of plasticity and therefore skeletal resilience (Blundon and Vermeij, 1983; Jarrett, 2008; Lord and Whitlatch, 2012; Sherker et al., 2017). The main point is that, by exposing hard-shelled prey to drilling predation on a daily basis, calcifying animals have had to deal with and respond to the effects of acidic dissolution, especially from the Late Mesozoic onward.

Bioerosion—the formation of tunnels or borings in hard substrata—is practiced by a wide variety of cyanobacteria, protists and animals. Although it is sometimes accomplished by abrasion or mechanical pulverization of hard materials, bioerosion is usually aided by acid secretion from specialized cells or glands. Unlike drilling, which affects only living targets, bioerosion is particularly intense in limestone, sedimentary rocks and the skeletons of dead organisms. Nevertheless, it also affects the skeletons of living organisms, which show various adaptations that deter penetration. An external organic shell layer (the periostracum) is effective against bioeroders, as are internal organic sheets between mineralized shell layers (Isaji, 1993; 1995). For exampke, rock- and shell-boring mussels (clade Mytilidae), which rely on acidic secretions to excavate the cavities in which they live, also have organic layers to protect their own shells from the acids they deploy (Owada, 2009). The depth and intensity of bioerosion have increased since the Cambrian, especially in nutrient-rich environments where food for suspension-feeding borers is abundant (Vermeij, 1987). Again, the point is that calcifiers exposed to acid-secreting bioeroders on a daily basis are well predisposed adaptively to combat ocean acidification. In particular, it is intriguing that the organic covering of most animal skeletons protects against acidified waters (Ries, 2011) but also against the more widespread and quotidian activities of bioeroders and drilling predators.

Recognizing the role these selective pressures play in deep resilience offers avenues for future research. Mineralogy and microstructure have been thoroughly examined in a descriptive way (Checa, 2018), but experimental work testing how various microstructures perform under ocean acidification is insufficient to make strong conclusions (Figuerola et al., 2021). There is some evidence that calcitic skeletons are more resistant to drilling (and bioerosion) than aragonitic ones (Gabriel, 1981), but how important this difference is in deterring drilling in nature is unclear. Organic layers may also be important—organic rich structures such as nacre are known for their strength—but they also take more energy to produce (Palmer, 1992). Studies using archival museum collections to study historical trends in calcifiers have identified multiple examples in molluscs and brachiopods where shifts in mineral layering and/or shell thickness have compensated for increased exposure to ocean acidification (Cross et al., 2018; Bullard et al., 2021; Telesca et al., 2021; Mayk et al., 2022). Finally, the ability of some calcifiers to regulate shell dissolution–such as those that perform bioerosion or those that dissolve parts of the shell to generate new shapes (Vermeij, 2020)–suggests that unknown genetic controls of decalcification exist in some lineages. How species are capable of performing these changes in mineralogy, and which groups are capable of performing them, are largely unknown. As a starting hypothesis, we predict that clades which have had to deal with and respond to the effects of dissolution linked to predation are more likely to be resilient to ocean acidification. Understanding how all these shifts in mineralogy, perhaps spurred on by predation, ultimately impact resilience should provide useful insight into which clades are at most risk to ocean acidification.

### Microbial interactions

Bacteria are the oldest (Allwood et al., 2006), the most diverse (Görgen et al., 2021), and perhaps the most prolific (Altermann et al., 2006) source of biogenic carbonates, yet their role in eukaryotic calcification is largely unknown. Most research on symbiotic partnerships as it relates to calcification focuses on photosynthetic algae (particularly the dinoflagellate clade Symbiodiniaceae) and their ability to enhance calcification in reef-building corals and other marine invertebrates (Gattuso et al., 1999; LaJeunesse et al., 2018). Photosynthesizers such as Symbiodiniaceae promote “light enhanced” calcification by providing energy to their host, as well as building blocks for the skeleton’s organic matrix (Gattuso et al., 1999; Bertucci et al., 2015). Although photosynthesis can stimulate calcification in a variety of algae and other taxa (Borowitzka, 1982; Rost and Riebesell, 2004), free-living Symbiodiniaceae are not known to mineralize. However, they will readily produce calcifying biofilms through relationships with bacteria, at least in the laboratory (Frommlet et al., 2015; 2018). This raises an intriguing possibility that, through a microbial symbiosis, Symbiodiniaceae were preadapted to form relationships with calcifying animals, and fits within a broader hypothesis that low calcium availability in Cretaceous-age oceans encouraged Symbiodiniaceae to become an endosymbiont (Malcolm and April 2012; Frommlet et al., 2015). If this hypothesis is true, we predict that these biofilms will readily form in natural environments, and that their production can be controlled by changes in seawater calcium ion concentrations, neither of which has been demonstrated so far. But if accurate, it offers another mechanism to help explain the decoupling of animal biomineralization and environment through the Mesozoic, and explain why corals as a group show little response to changing seawater chemistry over geologic time (Eichenseer et al., 2019). The potential for microbes to help mediate Symbiodiniaceae-host interactions is unlikely to be merely historical, and is beginning to get serious consideration (Matthews et al., 2020). The stabilizing effect of microbes on eukaryotic calcification may not even require Symbiodiniaceae as an intermediate. A diversity of photosynthetic cyanobacteria, for example, can produce amorphous calcium carbonate (Benzerara et al., 2014), a potentially universal building block of animal calcification (Gilbert et al., 2022). And while Symbiodiniaceae are restricted to the tissues of their coral host, coral skeletons harbor a diverse array of microbes, including cyanobacteria, which have the potential to enhance or weaken the calcified structure (Pernice et al., 2020). Marine photosynthesizers require CO_2_ and bicarbonate ions, which are expected to increase under ocean acidification. This means that increased photosynthesis could provide a buffer for calcifying organisms with photosymbionts (Ries et al., 2009).

An important and outstanding question is whether, and to what extent, calcification in non-photosynthetic animals might be aided by facultative or even obligate partnerships with calcifying bacteria (Vermeij, 2014). Many non-photosynthetic microbes produce carbonates (Görgen et al., 2021), and heterotrophic bacteria can be just as important as cyanobacteria in the lithification of stromatolites (Andres et al., 2006). Such microbes, residing in the site of calcification of the host, might aid in precipitating a template for the skeleton organic matrix to form. Microbial communities in calcifying microenvironments might also play a buffering role by more generally raising the alkalinity through their own metabolism, effectively counteracting the potentially deleterious pH environment of ambient water. The extrapallial fluid of the eastern oyster (*Crassostrea virginica*), for example, has a distinct microbiome, enriched in organisms that perform putative sulfate and nitrate reduction (Sakowski et al., 2020). Another possible role for microbes is in remote calcification, or biomineralization away from shell-secreting tissues and organs. Such remote calcification is documented in cephalopods (Checa et al., 2022) as well as the mucus of veneroidean bivalves (Taylor et al., 1999) and vermetid gastropods (Rezende et al., 2021). Mineralizing bacteria have been explicitly hypothesized to drive remote calcification in the latter two groups. We have performed experimental work on the chalky deposits of oysters, which offers another example of remote calcification, but so far have been unable to demonstrate a bacterial contribution (Banker and Vermeij, 2018; Banker and Coil, 2020; Banker and Sumner, 2020). However, chemical manipulation of the microbial community in juvenile oysters does cause a significant change in shell size, and a return to normal shell growth correlates with a reestablishment of the wildtype microbiome, which appears to be mediated by the oyster (Banker et al., 2022). Other scientists are also exploring microbiomes in marine calcifiers, although most of the work is similarly nascent, and focused on determining the taxonomy and distribution of microbes residing in calcifying fluids or skeletons (Li et al., 2014; Garate et al., 2017; Prazeres et al., 2017; Marcelino et al., 2018; Sakowski et al., 2020). Little is known about the function of these microbiomes, to the point that we do not currently know whether they help or hinder their host’s calcification during periods of stress. Microbial inputs to calcification is one of many exciting areas in microbiology, and clearly a lot more work is needed to determine whether they play any role in deep resilience.

## Conclusion

The fossil record provides evidence for deep resilience in the skeletons of marine organisms. This helps explain why near-term ocean acidification has little to no impact on many calcifiers. Comparative genetics may ultimately reveal a biomineralization toolkit shared by these calcifiers, but that is unlikely to explain deep resilience, at least within the paradigm of canalization. In addition to genetic work, research on the physiology and evolutionary history of species will be important to explaining resilience. We hypothesize that the increased resilience of calcified skeletons to environmental change has a strong biological component, expressed by intensifying selection from predators and by the evolution of partnerships with microbes. This has resulted in several mechanisms that have the potential to increase resilience, including the ability to manipulate shell mineralogy, the ability to protect the skeleton with organic covering, and the ability to regulate pH in specific microenvironments. Additional mechanisms are likely to be discovered through careful attention to patterns in the fossil record.

As important as the fossil record has been to our ideas, much of the future research into deep resilience–and, we suspect, climate change’s impact on calcifiers more broadly–will come from detailed studies of the sites of calcification. Calcification is a localized process; we need to understand the microenvironments where calcification is taking place, including any resident microbiome. Fine-scale sampling approaches will be needed to isolate calcifying fluids, and to identify different communities within skeletons, which have the potential to be highly heterogeneous (Marcelino et al., 2018). Perhaps the most important factor influencing deep resilience over time is an organism’s power budget, the amount of energy and time available to an organism to carry out the many competing functions of life (Vermeij., 2020). There have been demonstrated increases in maximum power over time in calcifying as well as other organisms including plants (Vermeij., 2020), implying that more resources have been devoted to the construction and maintenance of external skeletal protection over time. Energetic costs of calcification may be relatively low, and are calculated to only increase modestly (∼10%) under near-term ocean acidification scenarios (Spalding et al., 2017). It seems probable that different clades have evolved unique combinations of mechanisms to keep energetic costs for their skeletons low, thus creating a pattern of deep resilience. The added cost of calcification under acidic conditions may therefore be more easily borne by organisms with larger energy (or power) budgets, such as crustaceans. Uncovering those mechanisms should explain a great deal about the evolutionary trends seen in the fossil record, and help us predict which calcifiers will prove the most resilient in the oceans to come.

## Data Availability

The original contributions presented in the study are included in the article/supplementary material, further inquiries can be directed to the corresponding author.
